# *Candidatus* Syngnamydia Venezia, a Novel Member of the Phylum *Chlamydiae* from the Broad Nosed Pipefish, *Syngnathus typhle*

**DOI:** 10.1371/journal.pone.0070853

**Published:** 2013-08-12

**Authors:** Alexander Fehr, Elisabeth Walther, Heike Schmidt-Posthaus, Lisbeth Nufer, Anthony Wilson, Miroslav Svercel, Denis Richter, Helmut Segner, Andreas Pospischil, Lloyd Vaughan

**Affiliations:** 1 Institute of Veterinary Pathology, Vetsuisse Faculty, University of Zurich, Zurich, Switzerland; 2 Centre for Fish and Wildlife Health, Vetsuisse Faculty, University of Bern, Bern, Switzerland; 3 Institute of Evolutionary Biology and Environmental Studies, University of Zurich, Zurich, Switzerland; University of California, San Francisco, University of California, Berkeley, and the Children’s Hospital Oakland Research Institute, United States of America

## Abstract

*Chlamydia* are obligate intracellular bacteria and important pathogens of humans and animals. *Chlamydia*-related bacteria are also major fish pathogens, infecting epithelial cells of the gills and skin to cause the disease epitheliocystis. Given the wide distribution, ancient origins and spectacular diversity of bony fishes, this group offers a rich resource for the identification and isolation of novel *Chlamydia*. The broad-nosed pipefish (*Syngnathus typhle*) is a widely distributed and genetically diverse temperate fish species, susceptible to epitheliocystis across much of its range. We describe here a new bacterial species, *Candidatus* Syngnamydia venezia; epitheliocystis agent of *S. typhle* and close relative to other chlamydial pathogens which are known to infect diverse hosts ranging from invertebrates to humans.

## Introduction

Host-pathogen co-evolution is an evolutionary arms race driving the simultaneous evolution of pathogen virulence and host defenses. This interplay is especially tight between obligate intracellular microbes, such as members of the phylum *Chlamydiae*, and their eukaryotic hosts. The majority of empirical data on this phylum comes from studies of a single family, the *Chlamydiaceae*, whose members are major pathogens of terrestrial vertebrates, including humans. Due to their obligate intracellular biphasic lifestyle, *Chlamydia* cycle between an infectious particle, the elementary body (EB), and vegetatively replicating bodies (RBs) undergoing clonal expansion in specialised membrane-bound cytoplasmic organelles or inclusions. These features impose tight constraints on chlamydial evolution, which appears to be more influenced by recombination when different inclusions within a cell fuse, rather than by genetic exchange with other free-living bacteria [1].

Although molecular clock approaches have not been applied to the study of chlamydial evolution, the rate of genetic change in this group appears to be slow, given a high degree of synteny between different human strains of the species *Chlamydia trachomatis* or *Chlamydia pneumoniae* [1], [2], [3]. The evolutionary origins of the phylum *Chlamydiae* are old, associated with the rise of the cyanobacteria and plant life. However, it remains unclear to what extent the features of the family *Chlamydiaceae* are characteristic for the phylum as a whole. Answers are beginning to come through genomic studies into members of other families, notably the *Waddliaceae* [4], *Protochlamydiaceae* [5], [6], *Parachlamydiaceae* [7], *Criblamydiaceae* [8], [9] and most recently the *Simkaniaceae* [10]. Their genomes are intermediate in size (2.1–2.6 Mbp) between the *Chlamydiaceae* (0.9 Mbp–1.2 Mbp) and free-living bacteria such as *E. coli* (4.6 Mbp), but are comparable in size to the closest free-living relatives in the phylum *Verrucomicrobia* [11].

There appears to be a core set of genes shared by the phylum *Chlamydiae* [10], including cysteine-containing periplasmic and membrane proteins present in families known to be pathogens of land vertebrates (*Chlamydiaceae*, *Waddliaceae*, *Parachlamydiaceae* and *Simkaniaceae*), and which, at least in the *Chlamydiaceae*, are thought to be essential for maintaining membrane structural integrity in the absence of a peptidoglycan layer [12]. Some of these proteins are also associated with responses of the bacteria to a host humoral immune response [13], which could be a feature separating primary vertebrate chlamydial pathogens from chlamydial pathogens of unicellular organisms. In recent years, many novel members of the phylum *Chlamydiae* have been isolated from environmental aqueous sources using the highly successful amoebal co-culture method, leading to an inevitable bias in our understanding of their function. Indeed, there is a school of thought which proposes that free living amoebae may have provided an initial training ground for evolution of *Chlamydiae*, prior to their expansion into multicellular hosts and finally land-based animals and birds [7], [14], [15]. If this is the case, the question is who were the intermediate evolutionary hosts? Given the deep evolutionary history of *Chlamydia*, it follows that the major vertebrate hosts of the *Chlamydiae* prior to the emergence of land animals were fish.

The major chlamydial disease of fish is epitheliocystis, a term first coined by Hoffmann and colleagues [16] to describe infections of bluegill sunfish (*Lepomis macrochirus*) by bacteria ascribed to the “psittacosis-lymphogranuloma-trachoma group (*Chlamydozoaceae*)” based on appearance in transmission electron microscopy (TEM). The primary targets of this disease are epithelial cells of the gills and the skin, with the perinuclear inclusions forming cysts ranging in size from a few micrometers to many tens of micrometers. Epitheliocystis has been described in more than 50 fish species [17], [18], [19], with a wide range of morphologies infecting both juveniles and adults. Molecular data has only recently become available [20], [21], [22], [23], [24] and in only a few cases do we have both sequence information and ultrastructural data for infectious strains [20], [22], [24], [25]. This lack of information lies at the core of debates concerning the assignment of different ultrastructural morphologies to different chlamydial species and their putative developmental stages, a discussion that can only be resolved by a considerable expansion of our coverage of epitheliocystis, now further underlined by a recent report suggesting that intracellular bacteria other than members of the *Chlamydiae* can also cause epitheliocystis [26]. In addition, apart from *Ca.* Clavochlamydia salmonicola [22], [24], we have rarely found unequivocal morphological evidence for elementary bodies in epitheliocystis inclusions. This raises the question at what stage the biphasic life cycle, a central feature of the family *Chlamydiaceae* and for a long time part of the dogma of what “makes a *Chlamydia* a *Chlamydia*”, arose in the evolution of the phylum [18], [19], [24]. To explore such possibilities in greater depth, suitable marine vertebrate models of *Chlamydia* infection are required to facilitate the investigation of host-pathogen evolution.

Pipefish and seahorses of the *Syngnathidae* are the focus of evolutionary studies at both the genetic and behavioural levels in many parts of the world [27], [28], [29]. This research often involves the collection of fish from free-living populations, requiring basic data on their population genetic structure. The European broad-nosed pipefish (*Syngnathus typhle*) is one case in point, with a pan-European distribution of genetically distinct populations [30]. Northern populations of this species arose after the end of the last glacial maxima (ca. 20,000 years ago) and contemporary populations of this species are exposed to very different environmental conditions. Given the clear genetic differences among populations of *S. typhle* [30], their pathogens might be expected to show a corresponding genetic diversity, reflecting an extended period of host-pathogen coevolution. Pipefish are susceptible to various diseases [31], including epitheliocystis (Schmid-Posthaus, personal observations). As a first step towards establishing whether chlamydial infections in pipefish could offer an appropriate evolutionary model, we have begun the characterisation of epitheliocystis in pipefish collected from the Lagoon of Venice, one of the most genetically diverse populations of *S. typhle* [30]. The epitheliocystis agent we describe below has a number of attractive features, which have the potential to make it a model for *Chlamydia* research.

## Materials and Methods

### Sample Collection

*S. typhle* were collected by trawling (4 mm mesh) eelgrass beds in the Lagoon of Venice (45°13.86′N, 12° 16.59′E) in June and September 2011 and transported to the laboratory in 60 l tanks, with constant aeration. Animals were euthanized in buffered 3-aminobenzoic acid ethyl ester (MS 222®, Argent Chemical Laboratories, Redmont, USA) in filtered seawater and immediately dissected. Tissue samples for histopathological analysis were fixed in 10% buffered formalin in seawater. Gills were removed under aseptic conditions and examined under a dissecting microscope for lesions, including the presence of epitheliocystis. When present, cysts were dissected free of surrounding fine gill lamellar tissue, rinsed in several changes of sterile sea water and prepared directly for DNA extraction or immediately frozen in liquid nitrogen for later analysis. Gill lamellae from fish containing cysts were also fixed in 10% buffered formalin for histology or in 2.5% gluteraldehyde in 0.1 M sodium phosphate buffer, pH 7.5 for electron microscopy. Formalin-fixed gill samples were paraffin-embedded and sections of 3 µm thicknesswere stained with haematoxylin-eosin (H&E) for histopathological examination.

### DNA Extraction, PCR Amplification and DNA Sequencing of Epitheliocystis Positive Gills and Isolated Cysts

Genomic DNA was extracted from fresh or frozen gill tissue or from individual cysts using a commercial DNA extraction kit, according to the manufacturer’s instructions (DNeasy Tissue kit; Qiagen, Hilden, Germany). The presence of chlamydial DNA in the samples was subsequently determined by broad-range order *Chlamydiales*-specific 16S rRNA PCR, targeting the *Chlamydiales*-specific 280 bp 16S rRNA gene signature sequence [23] or the nearly full length 16S rRNA gene of 1500 bp, as previously described [20]. Negative controls (dH_2_O) were performed in triplicate. PCR products were visualised by UV transillumination (254 nm) following the separation of PCR products by agarose gel electrophoresis.

Freshly-amplified 16S rRNA gene PCR products were ligated into the pCR2.1-TOPO cloning vector (Invitrogen, Basel, Switzerland) and transformed into *E. coli* TOP10 chemically-competent cells (Invitrogen), according to the manufacturer’s instructions. 5–10 individual clones derived from a single cyst or from gill lamella containing multiple cysts were screened by restriction digestion and inserts were capillary sequenced on a 3130xl Genetic Analyzer (Applied Biosystems) using M13 forward (5′-CGCCAGGGTTTTCCCAGTCACGA-3′) and M13 reverse primers (5′-AGCGGATAACAATTTCACACAGGA-3′).

### Bioinformatics

Sequences were analysed with CLC Main workbench, version 6.8.1 (CLC bio Denmark), trimming and editing sequences after reviewing base-calls for accuracy. After alignments with ClustalW2 [32] or MUSCLE [33] on phylogeny.fr [34] using default settings, fasta files were generated. To examine the relationships between the sequences, phylogenies were constructed in MEGA5 [35]. Phylogenetic trees were inferred by the neighbour-joining (NJ) and the maximum-parsimony (MP) methods and tree reliabilities were assessed by 1000 bootstrap replicates [36]. Sequences were also identified using Blastn searches against the nucleotide collection of GenBank (www.ncbi.nlm.nih.gov) [37].

### Transmission Electron Microscopy (TEM)

Gill branches fixed with 2.5% glutaraldehyde in 0.1 M sodium phosphate buffer, pH 7.5 at 4°C were prepared for embedding into epoxy resin and for TEM according to standard procedures. Gill sections containing epitheliocystis lesions were selected from epoxy resin blocks using semithin sections (1 µm) stained with toluidine blue (Sigma–Aldrich). Ultrathin sections (80 nm) were mounted on copper grids (Merck Eurolab AG, Dietlikon, Switzerland), contrasted with uranyl acetate dihydrate (Sigma–Aldrich) and lead citrate (Merck Eurolab AG) and investigated using a Philips CM10 transmission electron microscope. Images were processed with Imaris (Bitplane, Zurich) and assembled for publication using Adobe Photoshop.

### Fluorescence In Situ Hybridisation (FISH)

*E. coli* TOP10 transformed with the full-length 1500 bp 16S rRNA gene fragment of fragments of *Waddlia chondrophila* [4] or *Ca*. Syngnamydia venezia (the major consensus amplicon, GenBank Accession No. KC182514, which we have named *Ca*. Syngnamydia venezia, see below) in the pCR2.1-TOPO cloning vector or with the empty vector were spotted out onto glass slides (coated with 0.01% poly-L-lysine) and used as positive or negative controls to establish the appropriate hybridization conditions, by increasing the formamide concentrations in 5–10% steps and decreasing the salt concentrations accordingly [38]. This was achieved using a Cy5-labelled chlamydiales-specific Chls-523 probe (S-O-Chls-0523-a-A-18∶5′ –CCTCCGTATTACCGCAGC- 3′, used with competitor (com Chls-0523) 5′-CCTCCGTATTACCGCGGC- 3′, as previously described by Poppert and colleagues [38] as well as a 5(6)-carboxyfluorescein-*N*-hydroxysuccinimide ester (FLUOS) 5′-labelled eubacterial oligoprobe (Eub338∶5′ –GCTGCCTCCCGTAGGAGT- 3′, binding site in *E. coli* 16S rRNA gene 338 to 355) [39] to use as a positive control on *E. coli* and the isolated inclusions. Based on these conditions, cysts, isolated in the same manner as for the sequencing, were incubated at 46°C with FLUOS-Eub338 and Cy5-Chls-0523, in hybridization buffer containing 40% formamide. Detection was by means of a confocal laser scanning microscope (CLSM, Leica TCS SP5, Leica Microsystems). FLUOS and Cy5 were sequentially excited with the 488 nm and 631 nm laser lines respectively, with emission signals collected between 495–570 nm and 640–700 nm. 3D image stacks were collected sequentially (to prevent green–red channel cross-talk) according to Nyquist criteria (voxel size, x:y:z = 277 nm:277 nm:294 nm), processed by deconvolution using HuygensPro via the Huygens Remote Manager v2.1.2 (SVI, Netherlands) and prepared for publication using Imaris 7.6.1 (Bitplane, Zurich, Switzerland).

## Results

**Figure 1:**
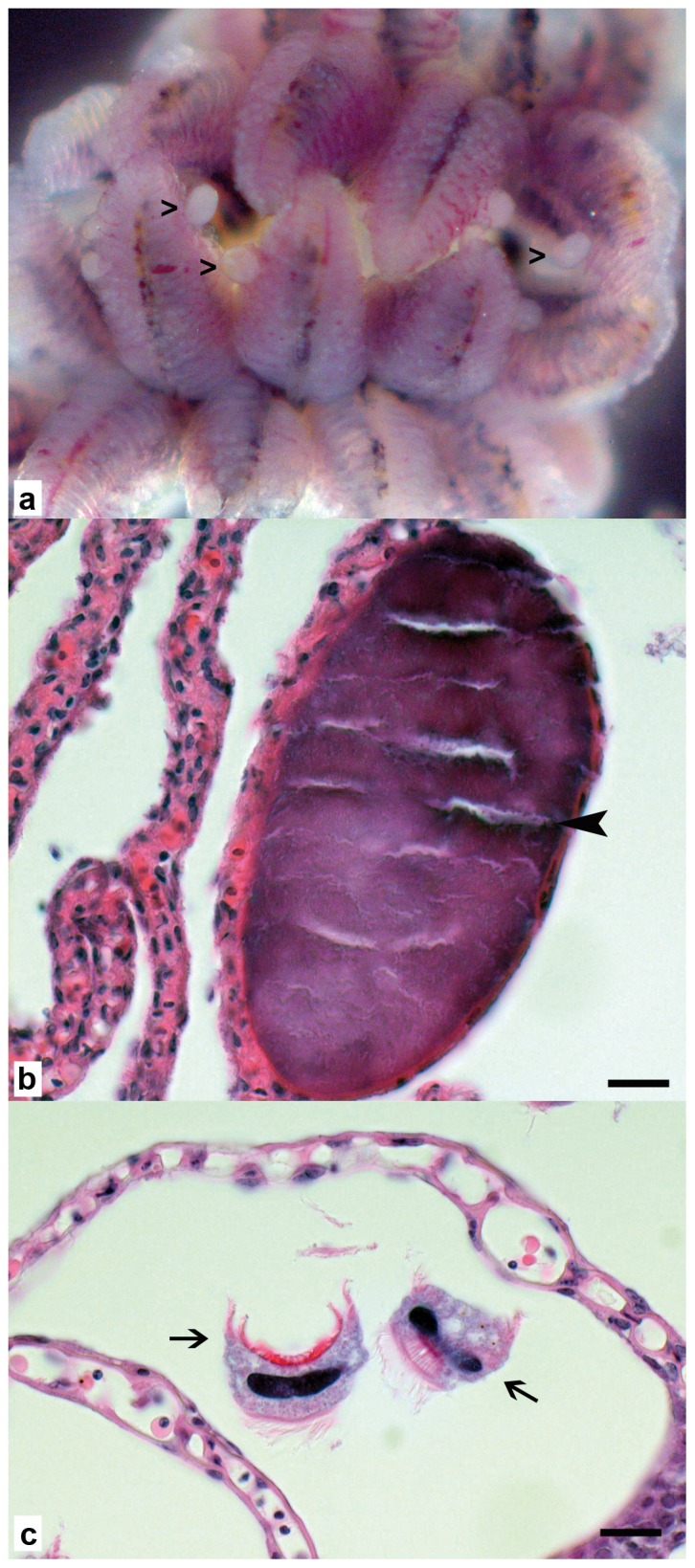
Wet mount and histology of pipefish gills. a: Wet mount in sterile sea water with numerous protruding epitheliocystis lesions clearly visible (open arrowheads). b: Gill lamella with focal intracellular cyst in epithelial cell, 40 µm long axis, with dark basophilic granular material (black arrowhead), the epithelium of affected lamella shows no pathological changes and no inflammatory reaction, scale bar = 10 µm. c: multiple *Trichodina* sp. between lamellae (arrows), no associated pathological changes, scale bar = 10 µm.

Broad-nosed pipefish (*S. typhle*) gills were prepared free from surrounding tissues using aseptic conditions under the dissecting microscope (Fig. 1). The epitheliocystis lesions could be readily seen as large (40–300 µm) oval, pale pearlescent-coloured cysts anchored to the gill lamellae. Histological examination of gill tissue revealed multiple intracellular cysts in enlarged epithelial cells. These cysts were not associated with any obvious changes in the gill epithelium or any inflammatory reaction. The cysts appeared to be the result of displacement of the cytoplasm and nucleus by a large (40–300 µm diameter), well-marginated vacuole containing basophilic granular material (Fig. 1b). There were also multiple 20–30 µm hat-like parasites between the lamellae, each with a basal rim of cilia and a central basophilic elongated nucleus (*Trichodina* sp.) (Fig. 1c).

**Figure 2:**
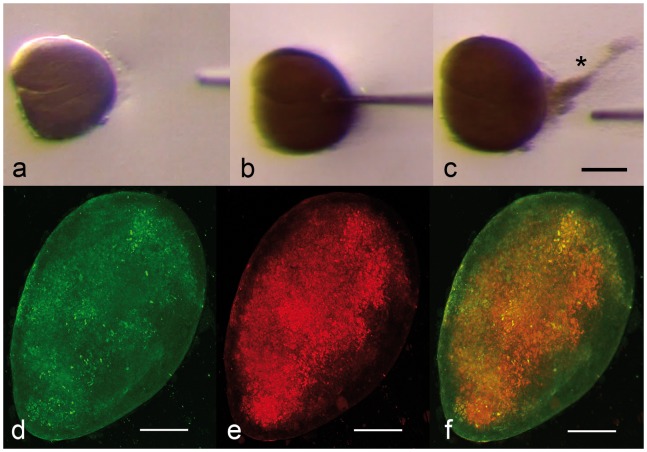
Wet mount and FISH of isolated inclusions. a-c: Wet mount of freshly isolated cyst in sterile sea water (a), punctured with a glass microinjection needle (b), releasing a thick cloud of bacteria (* c). FISH of isolated cyst, labelled with a eubacterial probe (green, d) and a *Chlamydiales*-specific probe (red, e), with the combined signals (f). The FISH images were collected as 3D image stacks by CLSM, deconvolved and the resulting image was compressed into a single 2D-image, shown here. Scale bars = 50 µm.

Using pairs of sterile injection needles, individual cysts were dissected free of associated lamellar tissue for analysis (Fig. 2). Puncturing an isolated cyst with a needle (Fig. 2a-c) released large numbers of fine granular particles (Fig 2c), consistent with bacteria of approximately 1–2 µm in size. To establish whether the bacteria within the cysts were members of the order *Chlamydiales*, we performed fluorescence *in situ* hybridisation (FISH) on isolated cysts using a general eubacterial oligoprobe, Eub338, labelled with carboxyfluorescein (FLUOS) [39] (Fig. 2d,f) and with an order *Chlamydiales*-specific oligoprobe, Chls-523, labelled with Cy5 [39] (Fig. 2e,f). As there was a limited supply of isolated cysts, conditions for hybridisation were established using *E. coli* TOP10 transformed with the full-length 16S rRNA gene 1500 bp fragments of *Waddlia chondrophila* [4] or of the major consensus amplicon (GenBank Accession No. KC182514, which we have named *Ca*. Syngnamydia venezia, see below) in the pCR2.1-TOPO cloning vector or with the empty vector. Both probes labelled the bacteria within the intact cysts, with the better labelling obtained with the *Chlamydiales*-specific Chls-523 probe, indicating that a sequence analysis of the isolated cysts should reflect this dominance.

Whole gill branches containing cysts and individual isolated cysts were prepared for molecular analysis from three individual fish collected in September 2011. Full-length chlamydial 16S rRNA gene PCR amplification of a whole gill branch containing multiple epitheliocysts from each fish as well as amplificates from individual isolated inclusions generated bands of ca. 1500 bp. These products were cloned using TOPO-TA and individual clones were purified and sequenced. A single full-length consensus sequence was obtained for a total of eleven clones. To test for overall uniformity, we applied the same screening procedure using the shorter 280 bp chlamydial signature sequence. Of a total of 117 individual clones sequenced, all but eight could be assigned to the same single consensus sequence identified in the eleven clones of the full length 16S rRNA gene. Of these eight, three clones were 100% identical to *Ca.* Clavochlamydia salmonicola. The remaining five sequences had 97–98% nucleotide identity to various uncultured *Chlamydiales*, listed in NCBI as unpublished and originating from diverse marine hosts including a sponge (*Callyspongia diffusa*), a mollusc (*Mya arenaria*) and Atlantic salmon (*Salmo salar*).

**Figure 3:**
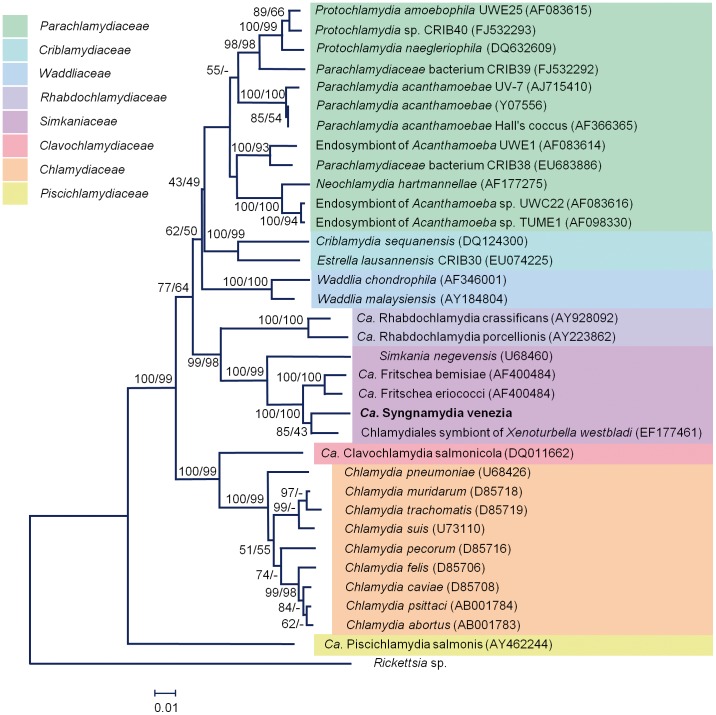
Phylogenetic tree of members of the phylum *Chlamydiae*, including epitheliocystis agents, calculated using NJ and MP analysis of the full length 16S rRNA gene sequence (1477 bp), using *Ricksettia* sp. as an outgroup. The percentage of replicate trees in which the associated taxa cluster together in the bootstrap test (1000 replicates) is indicated. Edited sequences were aligned in ClustalW2. Bootstrap values of 85%, and 43% in the NJ and MP trees, respectively, separated the new species from its closest relatives in the *Simkaniaceae* family.

The major consensus clade comprising 109 sequences, is novel and the corresponding full-length 1477 bp fragment is most closely related to a *Chlamydiales* symbiont of *Xenoturbella westbladi* [40] (96%, 1418/1479 bp), *Candidatus* Fritschea eriococci (94%, 1401/1484 bp) and *Candidatus* Fritschea bemisiae (94%, 1400/1487 bp) [41] and *Simkania negevensis* (93%, 1390/1492 bp) [10] (Fig. 3). Curiously, part of the sequence has 96% (1288/1345 bp) identity to an unpublished Chlamydiales symbiont of *Salmo salar* isolate D261006. According the criteria of Everett and colleagues [42], a 90% or higher 16S rRNA gene sequence homology places a chlamydial sequence within an extant family, with a cutoff of 95% for a new genus. On this basis, the novel *S. typhle* chlamydial endosymbiont likely represents a new genus, within the family *Simkaniaceae*.

**Figure 4:**
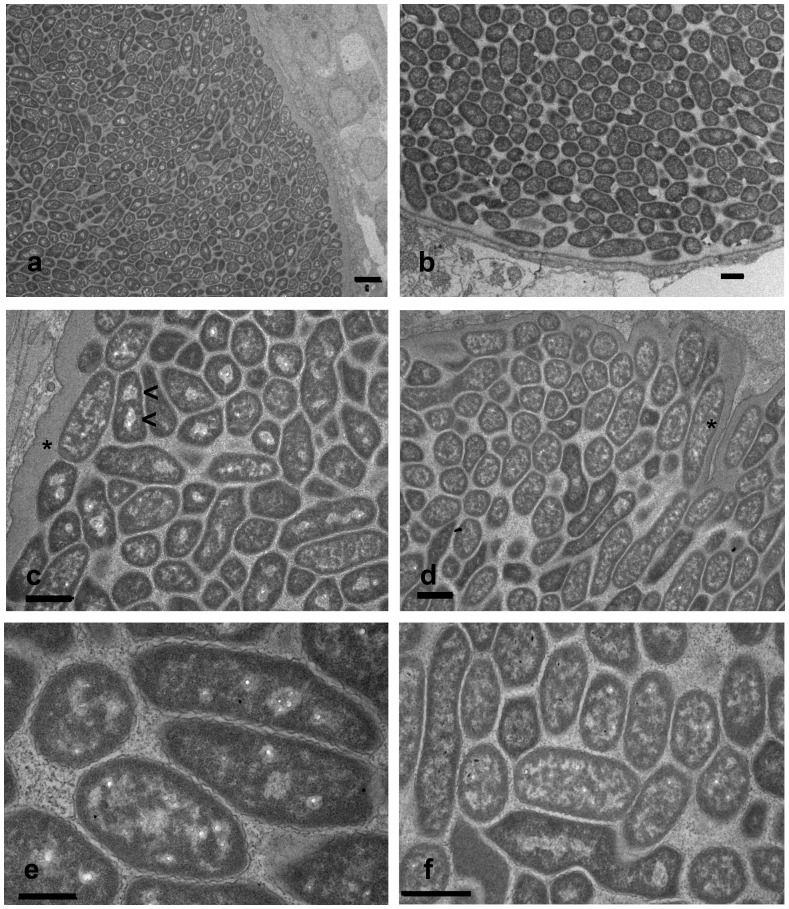
Transmission electron microscopy of epitheliocystis lesions showing features typical for *Candidatus* Syngnamydia venezia. Both a and b give an overview of the dense cell packing. In c and d, endosymbionts showing one or two electron-lucent regions (<), possibly representative of dividing bacteria, and maximally 3.4 µm (d, *) in length. The rippled outer membrane (e) as well as the angular forms (f) are typical characteristics. Scale bars = 1 µm.

To establish whether the novel chlamydial pathogen shares ultrastructural similarities with other members of the *Simkaniaceae*, we prepared TEM images of individual inclusions. All inclusions examined revealed a uniform population of particles (Fig. 4). Individual bacteria averaged 0.76±0.08 µm (SD, n = 42) in width and 2.17±0.44 µm (SD, n = 42) in length, when measured over several inclusions, although the longest bacterial bodies appeared to be dividing cells. In high-resolution images, individual bacteria exhibit an outer electron dense layer bounded by a rippled outer membrane. The bacterial cytoplasm was granular, often containing one or two clusters of electron-lucent regions, which did not appear to be delineated by a membrane. In contrast to the typical uniformly rounded forms of free-living gram-negative bacteria, the endosymbionts often exhibited tight angular forms, possibly indicative of a flexible outer membrane responding to the shapes or pressures exerted by the tight packing of adjacent bacteria in the inclusion. The epithelial cell inclusion membrane was moderately convoluted and 0.5–0.7 µm thick, delineated on the outside by a fine membrane, but with no clear inner membrane evident. We could find no evidence for spikes or bridges connecting the inclusion membrane to the endosymbionts, as we previously described for *Candidatus* Clavochlamydia salmonicola [24]. Bacterial particles appeared to be closely associated with, or even embedded within, the inclusion membrane. Pairs of electron-lucent clusters within endosymbionts, possibly indicative of dividing forms, could be found within bacteria throughout the inclusion. Similarly, longer (>2 µm) forms could be found well separated from, as well as adjacent to, the inclusion membrane.

## Discussion

The origins of the phylum *Chlamydiae* lie deep in the evolution of eukaryotes. Our knowledge of chlamydial biology stems almost entirely from a single family, the *Chlamydiaceae*, which are pathogens of land vertebrates, and little is known about how these pathogens have evolved from pathogens of fish, the only vertebrate hosts for the *Chlamydiae* from the Ordovician through until the Permian (488 - 299 million years ago). Epitheliocystis, the disease caused by members of the *Chlamydiae* in modern-day fish, has been described for well over 50 different fish species [17], [18], [19], although there are only a few cases linking morphological and molecular evidence. It has been suggested that the earliest *Chlamydiae* evolved to utilise the niche provided by single cell eukaryotes, in particular free living amoebae [7], [15], subsequently using the new skill set acquired to expand into multicellular animals. If this were generally the case, we would expect that a phylogenetic analysis of chlamydial endosymbionts of amoeba would reveal these to be the most ancient. Curiously, the most deeply branching member of the phylum *Chlamydiae* (Fig. 3) is an endosymbiont of fish, *Ca.* Piscichlamydia salmonis [15], [20]. In our own studies [19], [24], we could find no morphological evidence so far for a biphasic life-cycle of *Ca.* Piscichlamydia salmonis, raising the question as to whether this may be an evolved trait. To answer this, we need to improve our understanding of the biology of fish chlamydial endosymbionts. The results reported here are one step in this direction.

All previous efforts to isolate chlamydial endosymbionts from epitheliocystis lesions have proven unsuccessful. We have observed that members of the *Syngnathidae* (pipefish and sea horses) are susceptible to epitheliocystis, own observations and [21], including *S. typhle* collected from the Mediterranean sea, near Venice (this manuscript) and from the west coast of Sweden, near Göteborg [19], [30]. Due to the relatively large size of the epitheliocystis inclusions of *S. typhle* and their attachment to what appears to be a single epithelial cell of the fine secondary gill lamellae, without tissue thickening or hyperplasia, it was possible to dissect them cleanly from the gills under aseptic conditions for detailed characterization, allowing us to identify the aetiological agent by PCR-amplification and cloning of the full length 16S rRNA gene from isolated inclusions from three different fish using *Chlamydiae*-specific primers. The eleven clones sequenced differed at only a single site over their 1477 bp 16S rRNA gene sequence, indicating a single species. This result was confirmed by PCR amplification and cloning of the shorter 16S rRNA gene (∼280 bp) signal sequence from these samples, where 109 of 117 clones could be assigned to the same consensus sequence. Phylogenetic analysis places this species within the family *Simkaniaceae*, with 93–96% nucleotide identity to other members of this group, indicative of a new species [42]. The phylogenetic tree based on full-length 16S rRNA gene sequences agrees well with recent efforts to reconstruct relationships within the phylum *Chlamydiae* [8], [14], [20], [22], [23], [41], [43], [44]. A more thorough phylogenetic analysis would require analysis of additional genes, something we intend to pursue using next-generation sequencing technologies directly from individual inclusions, an analysis which is beyond the scope of the present report.

### Pleomorphic Morphology and Lack of Elementary Bodies

Given the phylogenetic position of the new sequence within the *Simkaniacea*, it was of interest to examine the ultrastructural morphology of this new chlamydial endosymbiont for comparison with published data for other members of the *Simkaniaceae*. Most striking is the pleomorphic, elongated form of approximately 0.75 µm width and 2 µm length, bounded by a rippled outer membrane and a granular electron-dense region extending into the bacterial cytoplasm, where it is interspersed with electron-lucent regions. Similar particles are found closely embedded within the inclusion membrane as well as dispersed throughout the inclusions. These cysts approximate flattened triaxial ellipsoids in form, so that a 50 µm×30 µm×20 µm inclusion would have an estimated volume of 15700 µm^3^ (volume = 4/3πabc, where a, b and c are the lengths of the half axis) and could contain 4,000–8,000 bacteria, depending on packing density.

We found no evidence of smaller electron-dense forms typical for EBs as have been described for other members of the *Simkaniaceae*. *Simkania negevensis*, originally isolated from cell cultures and subsequently shown to be a human respiratory pathogen especially common within the Negev region of Israel [45], [46], [47], was reported to exhibit both smaller electron-dense forms and larger replicative bodies, the latter similar in shape to the pleomorphic forms described here [46]. From the limited evidence available, a single figure from the original publication reproduced in Everett et al. [41], *Ca.* Fritschea bemisiae, an endosymbiont of insect gut epithelium, may also have similar replicating bodies to *S. negevensis* and possibly also condensed elementary bodies. A range of forms have been described for a *Chlamydiales* endosymbiont of phagocytic gastrodermal cells in the marine deuterostomes, *Xenoturbella westbladi* and *Xenoturbella bocki* [40], postulated to represent three different reticular bodies and two infectious or elementary bodies. The reticular bodies had in part a similar rippled outer membrane and a flexible outer membrane whose shape appears to adapt to the packing pressure of the surrounding bacteria.

### Conclusions

The *Simkaniaceae*, a single family within the phylum *Chlamydiae*, is pathogenic to hosts ranging from a primitive eukaryote described as being merely a “ciliated bag with epithelial epidermis and gastrodermis and a mouth” [40], through to insects, humans and now fish. To what degree the different morphologies of the *Simkaniaceae* are adaptations to markedly different cellular niches in such phylogenetically divergent hosts must await further studies, which will require their successful isolation for cultivation. The method described here for preparation of the inclusions, free from substantial remnants of host tissue, may aid in these efforts. Particularly opportune is the advent and annotation of the *S. negevensis* genome sequence [8]. This resource may well provide the scaffold for comparative genomics of members of the *Simkaniaceae* using direct sequencing technologies, enhancing our understanding of chlamydial evolution. Given this wide host range, the possibility of zoonotic transfer of members of the *Simkaniaceae* is not to be underestimated, and something which could increase the utility of this clade as a model for the study of host-pathogen evolution. Pipefish of the *Syngnathidae* feed largely on small crustaceans and plankton [48], [49] and are especially prevalent in the seagrass beds known to provide a critical habitat for many commercial fisheries and support a high diversity of invertebrate and fish life [50], [51]. These same areas also provide an important buffer between catchment areas from human settlements, land-based agriculture and near shore aquaculture and more pristine offshore marine or reef environments. Monitoring pathogen loads of important indicator species may be of use in efforts to sustainably manage these threatened biotopes and aid the parallel development of adjoining, sustainable aquaculture [52]. Future work will aim to further characterise this novel chlamydial endosymbiont, *Ca.* Syngnamydia venezia, and to take advantage of the existing knowledge on the genetic structure of European populations of *Syngnathus typhle* [30], to explore the genetic affinities between this putative pathogen and its host.

### Description of “Candidatus Syngnamydia venezia”

“*Candidatus* Syngnamydia venezia” [Syng.na.my’di.a L. F. n. *Syngnathus* name of host genus and *Chlamydiae* name of bacterial phylum; L. F. n. *Syngnamydia*, *Chlamydiae* originating from Pipefish; ve.ne.zi.a L. M. n. *Venezia,* lagoon from which the samples were collected].

The provisional taxon “*Candidatus* Syngnamydia venezia” contains intracellular bacteria that may infect gill cells of *Syngnathidae* in marine environments. Members of the taxon exhibit morphologies resembling other members of the *Simkaniaceae*, but as appears to be the case for at least one other *Chlamydiae* infecting fish hosts [19], [24], may not have a biphasic life cycle. The pleomorphic RBs are approximately 0.75 µm in width and 2 µm long, with longer forms of up to 3.5 µm likely replicating bodies shortly before division. In each RB, a rippled outer membrane bounds an electron-dense membrane-proximal layer surrounding a granular cytoplasm interspersed with electron-lucent regions, which can coalesce to form one or two clusters, the latter possibly indicative of dividing cells. The outer membrane appears to be quite malleable, the shape ranging from round to angular rods and appearing to fill the space provided by the stacking of the surrounding RBs. No clear EB or infectious particle with an electron-dense core was observed, raising the possibility that the RBs may themselves be able to initiate an infection, and lead to epitheliocystis in syngnathid hosts. The gill epithelial inclusions do not appear to cause tissue inflammation or hyperplasia and approximate flattened triaxial ellipsoids in form with a major axis ranging from 40–100 µm. The 16S rRNA gene of “*Candidatus* Syngnamydia venezia” has been deposited in GenBank (Accession No. KC182514). The 16S rRNA gene shows phylogenetic affinity towards the family *Simkaniaceae*.

## References

[1] Clarke IN (2011) Evolution of *Chlamydia trachomatis*. Ann N Y Acad Sci. 1230, E11–8. doi: 10.1111/j.1749-6632.2011.06194. 10.1111/j.1749-6632.2011.06194.x 22239534

[2] Mitchell CM, Hutton S, Myers GSA, Brunham R, Timms P (2010) *Chlamydia pneumoniae* is genetically diverse in animals and appears to have crossed the host barrier to humans on (at least) two occasions. PLoS Pathog 6: e1000903 doi:10.1371/journal.ppat.1000903 20502684 10.1371/journal.ppat.1000903 PMC2873915

[3] Mitchell CM, Hovis KM, Bavoil PM, Myers GS, Carrasco JA, Timms P (2010b) Comparison of koala LPCoLN and human strains of *Chlamydia pneumoniae* highlights extended genetic diversity in the species. BMC Genomics 11: 442–451. 20646324 10.1186/1471-2164-11-442 PMC3091639

[4] Bertelli C, Collyn F, Croxatto A, Rückert C, Polkinghorne A, et al (2010) The *Waddlia* genome: a window into chlamydial biology. PloS One 5(5): e10890. 20531937 10.1371/journal.pone.0010890 PMC2878342

[5] Horn M, Collingro A, Schmitz-Esser S, Beier CL, Purkhold U, et al (2004) Illuminating the evolutionary history of chlamydiae. Science 304: 728–730. 15073324 10.1126/science.1096330

[6] Casson N, Michel R, Müller K-D, Aubert JD, Greub G (2008) *Protochlamydia naegleriophila* as etiologic agent of pneumonia. Emerg Infect Dis 14: 168–172. 18258101 10.3201/eid1401.070980 PMC2600176

[7] Greub G, Raoult D (2004) Microorganisms resistant to free-living amoebae. Clin Microbiol Rev 17: 413–433. 15084508 10.1128/CMR.17.2.413-433.2004 PMC387402

[8] Thomas V, Casson N, Greub G (2006) *Criblamydia sequanensis*, a new intracellular Chlamydiales isolated from Seine river water using amoebal co-culture. Environ Microbiol 8: 2125–2135. 17107554 10.1111/j.1462-2920.2006.01094.x

[9] Lienard J, Croxatto A, Prod’hom G, Greub G (2011) *Estrella lausannensis*, a new star in the Chlamydiales order. *Microbes Infect.* . 13: 1232–1241. 10.1016/j.micinf.2011.07.003 21816232

[10] Collingro A, Tischler P, Weinmaier T, Penz T, Heinz E, et al (2011) Unity in variety–the pan-genome of the *Chlamydiae* . Mol Biol Evol 28: 3253–3270. 21690563 10.1093/molbev/msr161 PMC3247790

[11] Hou S, Makarova KS, Saw JH, Senin P, Ly BV, et al (2008) Complete genome sequence of the extremely acidophilic methanotroph isolate V4, *Methylacidiphilum infernorum*, a representative of the bacterial phylum *Verrucomicrobia*.Biol Direct. 3: 26. 10.1186/1745-6150-3-26 PMC2474590 18593465

[12] McCoy AJ, Maurelli AT (2006) Building the invisible wall: updating the chlamydial peptidoglycan anomaly. Trends Microbiol 14: 70–77. 16413190 10.1016/j.tim.2005.12.004

[13] Tan C, Hsia RC, Shou H, Haggerty CL, Ness RB, et al (2009) *Chlamydia trachomatis*-infected patients display variable antibody profiles against the nine-member polymorphic membrane protein family. Infect Immun 77: 3218–3226. 19487469 10.1128/IAI.01566-08 PMC2715660

[14] Collingro A, Poppert S, Heinz E, Schmitz-Esser S, Essig A, et al (2005) Recovery of an environmental *Chlamydia* strain from activated sludge by co-cultivation with *Acanthamoeba* sp. Microbiol 151: 301–309. 10.1099/mic.0.27406-0 15632447

[15] Horn M (2008) *Chlamydiae* as symbionts in eukaryotes. Annu Rev Microbiol 62: 113–131. 18473699 10.1146/annurev.micro.62.081307.162818

[16] Hoffman GL, Dunbar CE, Wolf K, Zwillenberg LO (1969) Epitheliocystis, a new infectious disease of the bluegill (*Lepomis macrochirus*). Antonie van Leeuwenhook 35: 146–158. 10.1007/BF02219125 4987372

[17] Nowak BF, LaPatra SE (2006) Epitheliocystis in fish. J Fish Dis 29: 573–588. 17026667 10.1111/j.1365-2761.2006.00747.x

[18] Vaughan L, Polkinghorne A, Schmidt-Posthaus H, Colorni A, Nufer L, et al. (2010) The astounding molecular and morphological diversity amongst novel members of the order *Chlamydiales*. Proceedings 12th International Human Chlamydia Congress. 357–360 (ISBN 0-9664383-3-7).

[19] Vaughan L, Fehr A, Walther E, Schmidt-Posthaus H (2013) Epitheliocystis and the importance of *Chlamydia*-related bacteria on fish health. in “Free-living amoebae: an evolutionary crib for emerging pathogens”, edited by G. Greub, Blackwell Publishing (in press).

[20] Draghi A, Popov VL, Kahl MM, Stanton JB, Brown CC, et al (2004) Characterization of “*Candidatus* Piscichlamydia salmonis” (order Chlamydiales), a *Chlamydia*-like bacterium associated with epitheliocystis in farmed Atlantic salmon (Salmo salar). J Clin Microbiol 42: 5286–5297. 15528727 10.1128/JCM.42.11.5286-5297.2004 PMC525185

[21] Meijer A, Roholl PJ, Ossewaarde JM, Jones B, Nowak BF (2006) Molecular evidence for association of chlamydiales bacteria with epitheliocystis in leafy seadragon (*Phycodurus eques*), silver perch (*Bidyanus bidyanus*), and barramundi (*Lates calcarifer).* . Appl Environ Microbiol 72: 284–290. 16391055 10.1128/AEM.72.1.284-290.2006 PMC1352285

[22] Karlsen M, Nylund A, Watanabe K, Helvik JV, Nylund S, et al (2008) Characterization of “*Candidatus* Clavochlamydia salmonicola”: an intracellular bacterium infecting salmonid fish. Environ Microbiol 10: 208–218. 17894816 10.1111/j.1462-2920.2007.01445.x

[23] Polkinghorne A, Schmidt-Posthaus H, Meijer A, Lehner A, Vaughan L (2010) Novel Chlamydiales associated with epitheliocystis in a leopard shark *Triakis semifasciata* . Dis Aquat Organ 91: 75–81. 20853744 10.3354/dao02255

[24] Schmidt-Posthaus H, Polkinghorne A, Nufer L, Schifferli A, Zimmermann DR, et al (2011) A natural freshwater origin for two chlamydial species, *Candidatus* Piscichlamydia salmonis and *Candidatus* Clavochlamydia salmonicola, causing mixed infections in wild brown trout (*Salmo trutta*). Environ Microbiol 14(8): 2048–57. 22176683 10.1111/j.1462-2920.2011.02670.x

[25] Mitchell SO, Steinum T, Rodger H, Holland C, Falk K, et al (2010c) Epitheliocystis in Atlantic salmon, *Salmo salar* L., farmed in fresh water in Ireland is associated with ‘*Candidatus* Clavochlamydia salmonicola’ infection. J Fish Dis 33: 665–673. 20629856 10.1111/j.1365-2761.2010.01171.x

[26] Toenshoff ER, Kvellestad A, Mitchell SO, Steinum T, Falk K, et al (2012) A novel betaproteobacterial agent of gill epitheliocystis in seawater farmed Atlantic salmon (*Salmo salar*). PLoS One 7: e32696. 22427865 10.1371/journal.pone.0032696 PMC3299688

[27] Mobley KB, Small CM, Jones AG (2011) The genetics and genomics of Syngnathidae: pipefishes, seahorses and seadragons. J Fish Biol 78: 1624–1646. 21651520 10.1111/j.1095-8649.2011.02967.x

[28] Rosenqvist G, Berglund A (2011) Sexual signals and mating patterns in Syngnathidae. J Fish Biol 78: 1647–1661. 21651521 10.1111/j.1095-8649.2011.02972.x

[29] Wilson AB, Orr JW (2011) The evolutionary origins of Syngnathidae: pipefishes and seahorses. J Fish Biol 78: 1603–1623. 21651519 10.1111/j.1095-8649.2011.02988.x

[30] Wilson AB, Veraguth IE (2010) The impact of Pleistocene glaciation across the range of a widespread European coastal species. Molec Ecol 19: 4535–4553. 20854410 10.1111/j.1365-294X.2010.04811.x

[31] Reimer LW, Hildebrand A, Scharberth D, Walter U (1994) *Anguillicola crassus* in the Baltic Sea: field data supporting transmission in brackish waters. Dis Aquat Org 18: 77–79.

[32] Larkin MA, Blackshields G, Brown NP, Chenna R, McGettigan PA, et al (2007) Clustal W and Clustal X version 2.0. Bioinformatics 23: 2947–2948. 17846036 10.1093/bioinformatics/btm404

[33] Edgar RC (2004) Muscle: A multiple sequence alignment method with reduced time and space complexity. BMC Bioinformatics 5: 113 doi: 10.1186/1471-2105-5-113 15318951 10.1186/1471-2105-5-113 PMC517706

[34] Dereeper A, Guignon V, Blanc G, Audic S, Buffet S, et al (2008) Phylogeny.fr: robust phylogenetic analysis for the non-specialist. Nucleic Acids Res 36: W465–W469 doi: 10.1093/nar/gkn180 18424797 10.1093/nar/gkn180 PMC2447785

[35] Tamura K, Peterson D, Peterson N, Stecher G, Nei M, et al (2011) MEGA5: molecular evolutionary genetics analysis using maximum likelihood, evolutionary distance, and maximum parsimony methods. Mol Biol Evol 28: 2731–2739. 21546353 10.1093/molbev/msr121 PMC3203626

[36] Felsenstein J (1985) Confidence-limits on phylogenies - an approach using the bootstrap. Evolution 39: 783–791. 28561359 10.1111/j.1558-5646.1985.tb00420.x

[37] Altschul SF, Madden TL, Schäffer AA, Zhang J, Zhang Z, et al (1997) Gapped BLAST and PSI-BLAST: a new generation of protein database search programs. Nucl Acids Res 25: 3389–3402. 9254694 10.1093/nar/25.17.3389 PMC146917

[38] Poppert S, Essig A, Marre R, Wagner M, Horn M (2002) Detection and differentiation of Chlamydiae by fluorescence in situ hybridization. Appl Environ Microbiol 68: 4081–4089. 12147510 10.1128/AEM.68.8.4081-4089.2002 PMC124059

[39] Amann RI, Binder BJ, Olson RJ, Chisholm SW, Devereux R, et al (1990) Combination of 16S rRNA-targeted oligonucleotide probes with flow cytometry for analyzing mixed microbial populations. Appl Environ Microbiol 56: 1919–1925. 2200342 10.1128/aem.56.6.1919-1925.1990 PMC184531

[40] Israelsson O (2007) Chlamydial symbionts in the enigmatic *Xenoturbella* (*Deuterostomia*). J Invertebr Pathol 96: 213–220. 17599345 10.1016/j.jip.2007.05.002

[41] Everett KD, Thao M, Horn M, Dyszynski GE, Baumann P (2005) Novel chlamydiae in whiteflies and scale insects: endosymbionts ‘*Candidatus* Fritschea bemisiae’ strain Falk and ‘*Candidatus* Fritschea eriococci’ strain Elm. Int J Syst Evol Microbiol. 55: 1581–1587. 10.1099/ijs.0.63454-0 16014485

[42] Everett KD, Bush RM, Andersen AA (1999) Emended description of the order Chlamydiales, proposal of *Parachlamydiaceae* fam. nov. and *Simkaniaceae* fam. nov., each containing one monotypic genus, revised taxonomy of the family *Chlamydiaceae*, including a new genus and five new species, and standards for the identification of organisms. Int J Syst Bacteriol 49: 415–440. 10319462 10.1099/00207713-49-2-415

[43] Corsaro D, Thomas V, Goy G, Venditti D, Radek R, et al (2007) ‘Candidatus *Rhabdochlamydia crassificans*’, an intracellular bacterial pathogen of the cockroach *Blatta orientalis* (Insecta: *Blattodea*). Syst Appl Microbiol 30: 221–228. 16934426 10.1016/j.syapm.2006.06.001

[44] Kostanjsek R, Strus J, Drobne D, Avgustin G (2004) ‘Candidatus *Rhabdochlamydia porcellionis*’, an intracellular bacterium from the hepatopancreas of the terrestrial isopod *Porcellio scaber* (Crustacea: *Isopoda*). Int J Syst Evol Microbiol 2: 543–549. 10.1099/ijs.0.02802-0 15023973

[45] Kahane S, Greenberg D, Friedman MG, Haikin H, et al (1998) High prevalence of “Simkania Z,” a novel *Chlamydia*-like bacterium, in infants with acute bronchiolitis. J Infect Dis 177: 1425–1429. 9593040 10.1086/517830

[46] Kahane S, Kimmel N, Friedman MG (2002) The growth cycle of *Simkania negevensis* . Microbiol 148: 735–742. 10.1099/00221287-148-3-735 11882708

[47] Friedman MG, Galil A, Greenberg S, Kahane S (1999) Seroprevalence of IgG antibodies to the chlamydia-like microorganism ‘Simkania Z’ by ELISA. Epidemiol Infect 122: 117–123. 10098794 10.1017/s095026889800185x PMC2809596

[48] Franzoi P, Riccato F, Franco A, Torricelli P (2004) Dietary differences in three pipefish species (*Osteichthyes*, *Syngnathidae*) related to snout morphology. Biologia Marina Mediterranea 11: 592–594.

[49] Oliveira F, Erzini K, Goncalves JMS (2007) Feeding habits of the deep-snouted pipefish *Syngnathus typhle* in a temperate coastal lagoon. Estuar Coast Shelf Sci 72: 337–347.

[50] Moksnes PO, Gullstrom M, Tryman K, Baden S (2008) Trophic cascades in a temperate seagrass community. Oikos 117: 763–777.

[51] Waycott M, Longstaff BJ, Mellors J (2005) Seagrass population dynamics and water quality in the Great Barrier Reef region: a review and future research directions. Mar Pollut Bull 51: 343–350. 15757733 10.1016/j.marpolbul.2005.01.017

[52] Segner S, Sundh H, Buchmann K, Douxfils J, Sundell KS, et al (2012) Health of farmed fish: its relation to fish welfare and its utility as welfare indicator. Fish Physiol. Biochem. 38: 85–105. 10.1007/s10695-011-9517-9 21681416

